# How multi-scale modeling can help examine social determinants of health and resulting disparities

**DOI:** 10.1371/journal.pcbi.1013284

**Published:** 2025-07-15

**Authors:** Kyoko Yoshida, Elsje Pienaar, Shalanda A. Bynum, Naomi Chesler, Mitchel J. Colebank, Jessie Heneghan, Nadra Tyus, Jasmine Miller-Kleinhenz, Bruce Y. Lee

**Affiliations:** 1 Department of Biomedical Engineering, University of Minnesota, Minneapolis, Minnesota, United States of America; 2 Weldon School of Biomedical Engineering, Purdue University, West Lafayette, Indiana, United States of America; 3 National Institute of Nursing Research, National Institutes of Health, Bethesda, Maryland, United States of America; 4 Edwards Lifesciences Foundation Cardiovascular Innovation and Research Center and Biomedical Engineering, Samueli School of Engineering, University of California, Irvine, Irvine, California, United States of America; 5 Department of Mathematics, University of South Carolina, Columbia, South Carolina, United States of America; 6 Public Health Informatics, Computational, and Operations Research (PHICOR), CUNY Graduate School of Public Health and Health Policy, New York City, New York, United States of America; 7 Center for Advanced Technology and Communication in Health (CATCH), CUNY Graduate School of Public Health and Health Policy, New York City, New York, United States of America; 8 Artificial Intelligence, Modeling, and Informatics, for Nutrition Guidance and Systems (AIMINGS) Center, Nutrition for Precision Health powered by All of Us Research Program, CUNY Graduate School of Public Health and Health Policy, New York, New York, United States of America; 9 John D. Bower School of Population Health, University of Mississippi Medical Center, Jackson, Mississippi, United States of America; Johns Hopkins University, UNITED STATES OF AMERICA

## Abstract

Social determinants of health (SDOH) are the conditions in which people live, work, and play, and the wider set of factors (e.g., social and economic systems and policies) that shape a person’s daily life. SDOH can differ significantly across communities and populations, having positive impacts for some and negative impacts for others. Ultimately, this results in differences in health and disease distribution, that are known as health disparities. Despite the known impacts of SDOH and calls to characterize, address, reduce, and eliminate health disparities, they persist and, in some cases, have worsened. To address this challenge, a session at the Interagency Modeling and Analysis Group Multiscale Modeling Meeting held on the National Institutes of Health campus from June 28th to 29th, 2023, considered potential ways that multiscale modeling can help characterize adverse SDOH and resulting health disparities. This perspective summarizes and synthesizes the session discussions as a call to action to promote and strengthen interdisciplinary science that merges the unique perspectives, experiences, and expertise of the SDOH and multiscale modeling scientific communities in the pursuit of knowledge to improve population health. Specifically, we identify current challenges and ways in which multiscale modeling is uniquely suited to address the challenges, as well as identify what is necessary to facilitate the successful application of multiscale modeling in SDOH research. We conclude with a discussion on the future of multiscale modeling in SDOH and health disparities research.

## Introduction

Social determinants of health (SDOH) are the conditions in which people are born, grow, learn, work, play, live, and age, and the wider set of structural factors shaping daily life conditions. These factors include social, economic, and legal policies and systems, and access to high-quality jobs, education, housing, transportation, food, healthcare, etc. SDOH have profound and sustained influences on health, functioning, and quality of life across diseases, conditions, and stages of life [1]. Different communities and populations have different SDOH exposures; some exposures are adverse and hamper health, while others are positive and thus promote health. Despite numerous calls to characterize, address, reduce, and eliminate health disparities, they persist and, in some cases, have widened largely due to the inattention to SDOH as the underlying causes of adverse health outcomes. SDOH cross multiple levels, temporal and spatial scales, and have complex and far-reaching direct and indirect consequences. Thus, traditional study methods alone are insufficient for characterizing SDOH effects, posing a significant challenge in understanding SDOH impacts on health.

Multi-scale computational modeling is a powerful set of approaches, methods, and tools that may help characterize and assess health disparities (HD) by quantifying the impact of complex SDOH interactions across scales. Computational modeling entails constructing computer-based representations of the components and processes comprising a complex system. Such modeling is multi-scale when these representations cross different spatial and temporal scales. For example, time scales can range from minutes to decades, while spatial scales can range from small (molecular or cellular scales) to large (environmental or societal scales). Although multi-scale models (MM) have been used to understand associations between adverse SDOH and health outcomes at the population scale [2,3], their potential to examine and assess the impact of SDOH on health and disease at the individual, biological scale remains largely untapped. A session at the Interagency Modeling and Analysis Group Multiscale Modeling Meeting held on the National Institutes of Health campus in Bethesda, Maryland, from June 28th to –29th, 2023, considered some potential ways MM can characterize the mechanistic biological impacts of SDOH and resulting HD. The session brought together MM and SDOH experts from academic and federal agencies to identify key opportunities and challenges to leverage MM to understand the underlying causes of poor health outcomes, which are often steeped in SDOH influences. This perspective summarizes and synthesizes the session discussions as a call to action for the SDOH and MM communities to collaborate and bridge scientific gaps to increase our understanding of SDOH impacts on individual and population health.

## The impact of SDOH and resulting disparities are multi-scale issues

Factors that influence our health can span from the societal and community levels to the cellular and molecular levels. These factors are referred to as ‘multi-level’ in social and behavioral science, and ‘multi-scale’ in mathematical and computational modeling fields.

To fully understand the health impacts of SDOH and their influences at the biological (i.e., organ, tissue, cellular, and subcellular) scales, a multi-scale framework may be necessary. As we consider this framework, we need to recognize that disparities at the organ, tissue, and cellular levels stem from adverse SDOH reflective of living within health-inhibiting conditions and systems [4]. Thus, disparities in health outcomes are *not* purely associated with biological mechanisms, but rather reflect complex interactions between SDOH exposures and our biological responses to these SDOH across multiple scales.

Some examples of the multi-scale nature and analysis of SDOH from the societal to individual scales include an agent-based model (ABM) that quantifies the impact of neighborhood crime (community-scale) on leisure-time physical activity rates (individual behavior-scale) and obesity (physiology/biology-scale) among African American women in Washington, DC [5] and an ABM that simulates the impact of placing point-of-purchase sugar-sweetened beverage (SSB) warning labels as a policy measure (societal- and community-scales) on SSB purchases (individual behavior-scale) and subsequently adolescent overweight and obesity prevalence (physiology/biology-scale) [6].

Breast cancer provides an illustrative example of multi-scale SDOH-HD impacts from societal to molecular scales [7,8]. Although well-studied in biomedical research, many factors and interactions that drive breast cancer *disparities* remain unknown [9]. Starting at the highest level of influence [10], **societal** factors, including organization and quality of healthcare (e.g., time to treatment and follow up), healthcare policies and regulations (e.g., private insurance patients have better outcomes) [11], and residential segregation [12] are associated with breast cancer mortality. At the **community** level, neighborhood disadvantage affects the breast cancer stage at diagnosis and survival rates [12]. At the **individual** level, socio-economic status impacts breast cancer risk, time to diagnosis, and mortality [12]; and transportation access affects the time to treatment initiation [13]. At the **organ/tissue** level, SDOH are associated with more aggressive tumor biology [14–16] as well as elevated inflammation and macrophage recruitment to the tumor micro-environment in African American women compared to European American women [7]. At the **cellular** level, SDOH manifest as more tumor-permissive macrophages in Black women with breast cancer compared to non-Black women [17]. Finally, at the **molecular** level, the BRCA1/BRCA2 gene mutation is a risk factor mainly identified in European American women [7]. DNA methylation, strongly influenced by SDOH, namely environmental factors, is associated with cancer and is increased in African American women [18,19] and women living in neighborhoods with adverse social and environmental exposures [18,19]. In summary, the disparate breast cancer outcomes between Black and white women cannot be solely attributed to differences in ancestry, and there is increasing evidence of SDOH-HD as underlying causes of breast cancer incidence, progression, and survival [20,21]. These examples illustrate the multi-scale nature of SDOH on health and health disparities.

## Challenges to characterizing and assessing the biological impact of SDOH

Table 1 lists some challenges to characterizing and assessing the biological health impacts of SDOH. One major challenge is that the biological effects of SDOH involve factors that are multi-scale and multifactorial. For example, social, educational, and economic conditions affect the ability of individuals to receive and seek care (individual level) [22], stemming from hindered access to quality education or jobs impacting health literacy and equitable care (population level). Further, the “social causation” hypothesis suggests that social interactions directly affect health outcomes and physiological function (organ or cell level) [23]. This provides evidence of a population-to-individual-to-organ level link that requires more comprehensive datasets and modeling infrastructures that can handle these nested processes. The time-lag between the initial social exposure and later biological effect (i.e., multiple temporal scales) is another challenge, as observable phenotypes may take months, years, or generations to manifest. Isolating each determinant can be difficult [24]. For instance, the “neuroimmune network hypothesis” [25] posits early-life adversity causes peripheral inflammation and increased neural stress, contributing to elevated insulin resistance, adiposity, and emotional health problems later in life. Early-life psychological stress causes dendritic atrophy in middle-aged animals [26], yet distinguishing whether other cofactors (e.g., chronic stress or diet) contribute to cognitive risk remains a challenge [27]. Early-life adverse SDOH can significantly disrupt key life-long biological and developmental pathways [28,29]. Thus, understanding the temporal impact of early-life adversity is difficult with human observational studies. Finally, SDOH is not isolated to one location, but can propagate to others. For example, individuals may relocate to seek better job opportunities and healthcare, which can improve or exacerbate SDOH in the new location. These complex interaction networks are difficult to quantify and disentangle with observational or experimental approaches alone.

**Table 1:** Major challenges in characterizing and assessing the impact of SDOH on health and health outcomes.

Challenge	Description	How Multi-scale Models (MMs) Can Help Overcome Challenge
**Complex System of Factors and Mechanisms**		
*Multi-scale and multifactorial factors involved*	Multiple factors crossing scales (population to subcellular) makes isolating impact of individual factors on outcomes challenging.	Build system from bottom-up to represent/ account for factors across scales Interrogating individual and synergistic effects of various factors to determine key drivers and how parameters at one scale impact another
*Time lag between cause and effect*	Translating SDOH to observable phenotypes can take months or generations.	Account for time scales, where each scale can change independently Simulate over defined lengths of time (e.g., day, month, years) Develop frameworks that can account for interdependent, temporal multiscale phenomena
*Causes in one location can affect SDOH in another location*	Causal factors of SDOH cross multiple spatial scales (e.g., community, region) and effects are not isolated to just one location and can be far-reaching.	Turn specific model relationships on/off, run “what-if” scenarios to test cause and effect hypotheses Reveal how different scales interact Explicitly incorporate and couple across scales to identify key intervention points with greatest impact
**Obtaining the Right Data**		
*Data can be biased or have important factors missing*	Health data can be biased (omitted variables, human bias, etc.) and gaps in collected data inevitably exists, including key measurements.	Run sensitivity analyses to identify key parameters (especially those with unknown or limited data) driving outcomes, thus identify high priority data to collect. Simulate unknown factors to generate output, which can be designed into testable hypotheses. Simulate “what-if” scenarios to test specific hypotheses related to factors that contribute to ailments in specific populations As data become available, can be updated with new data and estimates can be validated
**SDOH Cross Different Siloed Sectors and Perspectives**		
*Many researchers and decision makers work exclusively within one or a few scales*	Individual researchers tend to focus on the scale(s) where their output of interest occurs.	Bridge scientific fields, scales, and datasets. Expand translational impact of datasets to be more than sum of parts. Serve as bridges between scientific communities to quantify and translate data across scales.

Second, there are significant challenges in obtaining unbiased and accurate data in SDOH research. For example, data bias can be found in ancestry (e.g., genomics), demographics, socioeconomics, or methodological issues (e.g., how disease is measured). Researchers may create bias unintentionally (54) and the range of scales and the limitless data that could be collected inevitably lead to gaps in the necessary data to assess the biological impact of SDOH. Metrics tend to be biased towards overrepresented groups and may be less useful or applicable to underrepresented groups. Thus, assessments of how SDOH manifest into physiological or biological observations may underestimate the impact on populations. MMs may overcome these obstacles by encoding physiological hypotheses about a person’s general health and then examining whether the data being provided are consistent or inconsistent with the modeling framework. Moreover, MMs may allow for *in silico* testing of whether interventions may correct an individual’s trajectory, and then validate these findings to follow up data. A benefit to the MM framework is the mechanisms of population, individual, or sub-individual physiology are encoded and available even when data is limited.

Finally, multiple disciplines and study/analysis methodologies (genetics, biology, physiology, epidemiology, etc.) historically tend to operate exclusively within one or a few scales. This isolation of scales hampers our ability to quantitatively assess the broader picture of how SDOH influences and affects multiple scales. This limitation can be overcome with MMs, which encode multiple scales within a single simulation framework and can be leveraged with limited data sources at single spatial or temporal scales.

## How could multi-scale modeling help address these challenges?

For each challenge noted above, Table 1 lists ways MMs could help address these challenges. Many disciplines utilize MMs, including material science [30,31], fluid mechanics [32], biology [33], and epidemiology [34]—including the recent COVID-19 epidemic [35]. In application to SDOH-HD, researchers have used MM to assess US geographic variation in the factors associated with breast cancer mortality [2], evaluate the sociodemographic determinants of COVID-19 incidence rates in Oman [3], and develop an influenza forecasting tool at the state, regional, and national scales [34]. More recently, the study by Vodovotz and colleagues incorporated aspects of societal stress, inflammation, and intervention to address the multi-scale links in chronic stress [36]. The study provided interpretable links between external stimuli at a population level with their embedded biological processes at an individual level, key for MM for SDOH-HD. One of the key advantages of MMs is the ability to handle and integrate multimodal data. Data-driven models (i.e., top-down models) use dataset(s) with inputs and outputs of interest and define equations or algorithms to extract associations, correlations, and trends between certain factors and outcomes. These models can generate insights into complex relationships but do not necessarily capture cause and effect. In contrast, mechanistic models (i.e., bottom-up) aim to rebuild systems of interest and represent the mechanisms and causal pathways involved [37–39]. Traditional modeling approaches can be applied to MMs (i.e., sensitivity analyses) to identify key parameters to guide data collection or run “what-if” scenarios to generate testable hypotheses. These examples, and MM applications listed in Table 1, highlight the emergence and potential impact of MMs in SDOH-HD research.

## What is needed to facilitate the use of multi-scale modeling (MM) in SDOH research?

With SDOH’s impact on HDs being inherently complex and multi-scale, the potential for MM to advance our understanding and ability to address them is immeasurable. To advance MM in SDOH research, many of the previously described challenges, like an underdeveloped workforce, scientific siloing, lack of collaborations with the local, non-academic community, and lack of funding for example will need to be addressed. Table 2 shows what is needed to facilitate the use of MMs to address SDOH along with specific ways that they can be done.

**Table 2:** What is needed to facilitate the use of multi-scale modeling in SDOH research?.

Recommendation	How to Implement
**Develop the Workforce**	
*Incorporate multi-scale modeling (MM) into existing curricula and training programs*	Include courses on MM in all relevant degree programs’ curricula/requirements, ranging from undergraduate to graduate programs (e.g., master’s and doctorates in public health, sociology, urban planning, environmental health, etc.) Establish minor/major in MM
*Establish new MM training programs for different career levels*	Create degree and certificate programs for undergraduate, graduate, and mid-career levels Develop programs inherently interdisciplinary, including instruction/instructors from biophysical, clinical, mathematics, sociology, environmental health, and ethical sciences
*Establish new MM positions at different agencies and institutions*	Establish dedicated positions in MM at various public health and policy-related institutions to spearhead MM projects to address social determinants of health and health disparities (SDOH-HD) Design MM positions to establish pipeline of MM research projects and transdisciplinary collaborations
**Build Networks Across Disciplines and Communities**	
*Include SDOH/health equity experts on MM projects*	Develop collaborations with health equity experts on MM projects to synergize/build from base of work already underway in the field Invite SDOH-HD experts to educate MM community regarding SDOH parameters/factors, initiatives and funding opportunities, and how MM can support ongoing efforts
*Build transdisciplinary research networks*	Build relationships and collaborative research projects with individuals across disciplines, expertise, and skill sets (e.g., experts from biophysical, clinical, mathematics, social, environmental, and ethical sciences) to develop research network with range of different perspectives Research network should include modelers and SDOH/health equity experts
*Incorporate the perspectives of those most impacted by health inequities in MM projects*	Engage meaningfully with communities most impacted by SDOH-HD, using an open mind set and avoiding assumptions Build in perspectives of those most impacted by health inequities into research design Consider how comparison group is being defined/used for comparisons in simulations, including the research question and possible interventions [40]
*Build networks across researchers and communities*	Connect MM researchers from various fields/disciplines with local, non-academic communities Develop meaningful partnerships among diverse groups focused on building trust and open communication to guide research questions
**Increase Data Availability**	
*Identify data gaps*	Look for data that adequately represents ancestry of population being studied and, where appropriate, includes individual-level SDOH data, area-level characteristics, and exposures over various time-points Conduct a study to see what data is needed (see previous bullet) and of that data, what is available, to identify gaps
*Create databases that are accessible to researchers*	Establish online databases that pull in various datasets relevant to MM-SDOH-HD research projects and ensure its accessibility to MM researchers, SDOH experts This will require dedicated time, resources, and collaboration to prioritize and implement.
**Translate MM and its Output to Decision Making**	
*Get decision makers more acquainted with MM and its use/how it can help them make decisions*	Hold conferences, meetings, events, etc. with aim of introducing MM to decision makers in policy, public health, and industry Use lay terms, clear examples to show usefulness of MM to address complex questions in health/public health
*Incorporate decisions makers and those affected into the MM process*	Bring decision makers on board early in process to get buy-in and feedback to make sure research findings will be translatable into implementable projects, policies or interventions
*Use language that is easily understood by various audiences*	Ensure language used to describe MM and its findings is widely understandable and accessible to lay people, decision makers, people from different communities
*Display information in easy-to-understand visualizations*	Leverage breadth of communication tools, including graphic formats and visualizations, to convey information clearly to various audiences
*Consult communications experts to help translate MM to different communities*	Draw upon expertise of communications field to bridge gap with communicating MM findings to different groups (e.g., government officials, community organizing groups)
*Creating new venues for publishing and presenting*	Devote special issues of existing journals to MM approaches to address SDOH-HD Create new journals specializing in using MM to address SDOH-HD Establish conferences to allows researchers to present results and interact
**Increase Funding and Resources**	
*Establish new funding mechanisms that specifically support the use of MM to address SDOH-HDs*	Provide evidence of value of MM in the field Develop funding calls and conferences specifically geared towards these issues to spark conversation, new funding calls, and/or new collaborations.
*Establish shared resources that can facilitate MM of SDOH-HDs*	Establish computational resources and communal workspaces in disadvantaged communities to encourage researchers and other community groups to meet and work together in person These spaces should include computer clusters and access to cloud time

### Develop the workforce

For MM to be used to address SDOH, there needs to be more multi-scale modelers who are knowledgeable about SDOH, more SDOH researchers who can utilize MM, and more people in general who are better consumers of MM, meaning they understand the uses/benefits of MM and what is included as inputs, and are able to interpret the outcomes. Table 2 shows what actions can be taken to develop the workforce of MM in SDOH.

### Build networks across disciplines and communities

Since as indicated earlier, many different disciplines and communities remain separate and siloed, there needs to be active effort to break down the barriers and foster cross-disciplinary and cross-community collaboration on MMs. Table 2 lists some recommended efforts.

### Increase data availability

as indicated earlier, data gaps remain an issue. But first as Table 2 indicates, these gaps need to be fully identified. This can be done through a range of different studies including MM studies with sensitivity analyses determining the impact of better knowing the values of different parameters. When identifying data, it’s important to understand the biases that may be built into them (e.g., if the available longitudinal studies on the risk for breast cancer include participant samples drawn from largely upper-middle class, white populations, the resulting data will be missing key mechanisms and information, not be representative of other groups of people, and thus not generalizable due to these inherent biases in the data).

### Translating MM and its output to decision making

To increase the impact of MM studies, the findings need to be available and readily interpretable by decision makers at all levels, ranging from clinicians taking care of individual patients to administrators in charge of health systems to policy makers to the general public. Table 2 lists a set of ways that this can be done.

### Increase funding and resources

As with any scientific approach, to be done well MM requires at least adequate resources. A challenge is that many funding opportunities in the biomedical and health space are very focused on particular diseases or body parts when most real-world problems encompass a whole system of factors, processes, and effects. Moreover, a lot of these available resources are dedicated to specific content areas rather than methodologies that can address multiple content areas at a time. Table 2 describes ways to change this situation.

Naturally, many of these steps will require partnering among different disciplines and resources. The changes will likely take time, and dedicated investments are necessary for the benefits to come to fruition.

## The future of MM in SDOH research?

The use of MM to assess SDOH is at a critical inflection point. More data are available across different scales, including big data, especially with the advent of different wearables and data from social media and other Internet use [41]. The availability of sophisticated modeling approaches continues to grow as the available computational power enables the development of larger and more complex models and faster simulation times. Together, the use and value of MM in assessing SDOH are poised to grow by leaps and bounds.

Additionally, new developments are on the horizon. Artificial intelligence approaches can interface with and further expand the capabilities of MM. For example, a machine learning technique, *physics-informed neural networks* (PINNs), can expedite simulation time for expensive MM [42], eliminating the computational bottleneck often ascribed to MM. Other machine learning techniques can help identify missing biophysics processes in mechanistic models, providing a synergistic approach for machine learning-enhanced MM [43]. As always, artificial intelligence and machine learning should be used thoughtfully and with caution to avoid embedding and amplifying systemic biases [44–47].

Finally, effective communication and dissemination across various digital and multi-media platforms will improve MM access, understanding, and use. Developing user-friendly interactive interfaces and descriptions of such models can provide access to a wider range of users. Enhancing the accessibility of MM to multidisciplinary researchers could bolster its use for assessing SDOH. While assessing SDOH will be a great accomplishment, the goal is to use these SDOH assessments to enhance and scale up interventions, policies, and programs that reduce and someday eliminate health disparities. To that end, collaborations across fields/expertise areas will be required to make the necessary policy, law, educational, income, etc. changes effective and equitable.
